# Production of nutrient‐enhanced millet‐based composite flour using skimmed milk powder and vegetables

**DOI:** 10.1002/fsn3.777

**Published:** 2018-11-18

**Authors:** Gerald Tumwine, Abel Atukwase, Gaston A. Tumuhimbise, Francis Tucungwirwe, Anita Linnemann

**Affiliations:** ^1^ School of Food Technology, Nutrition and Bioengineering College of Agricultural and Environmental Sciences Makerere University Kampala Uganda; ^2^ Value Addition Institute Kampala Uganda; ^3^ Department of Agrotechnology and Food Sciences Subdivision of Food Quality and Design Wageningen The Netherlands

**Keywords:** germination, Millet flour, skimmed milk, vegetables

## Abstract

The aim of this study was to develop a nutrient‐enhanced millet‐based composite flour incorporating skimmed milk powder and vegetables for children aged 6–59 months. Two processing methods were tested to optimize nutrient content and quality of millet‐based composite flour, namely germination for 0, 24 and 48 hr and roasting at 80, 100, and 140°C. The amount of ingredients in the formulation was determined using Nutri‐survey software. Germinating millet grains for 48 hr at room temperature significantly (*p* < 0.05) increased protein content (9.3%–10.6%), protein digestibility (22.3%–65.5%), and total sugars (2.2%–5.5%), while phytate content (3.9–3.7 mg/g) decreased significantly (*p* < 0.05). Roasting millet grains at 140°C significantly (*p* < 0.05) increased the protein digestibility (22.3%–60.1%) and reduced protein (9.3%–7.8%), phytate (3.9–3.6 mg/g), and total sugar content (2.2%–1.9%). Germinating millet grains at room temperature for 48 hr resulted in millet flour with the best nutritional quality and was adopted for the production of millet‐based composite flour. Addition of vegetables and skimmed milk powder to germinated millet flour significantly (*p* < 0.05) increased the macro‐ and micronutrient contents and the functional properties of millet‐based composite flour. The study demonstrated that the use of skimmed milk powder and vegetables greatly improves the protein quality and micronutrient profile of millet‐based complementary foods. The product has the potential to make a significant contribution to the improvement of nutrition of children in developing countries.

## 1 INTRODUCTION

Millets are nutritionally rich and occupy an important place in the diet of people in many regions of the world (Jaybhaye, Pardeshi, Vengaiah, & Srivastav, 2014). In East Africa, especially Uganda, millet grains are the most important source of food for children aged 6–59 months (Shively & Hao, 2012; UBOS and WFP, 2013). As one of the most important drought‐resistant crops in Uganda, millets are widely grown in most parts of the country, especially the southwest, east, and northern regions (Adhikari, Nejadhashemi, & Woznicki, 2015). Millet serves as a major food component in various traditional foods and beverages such as bread and porridges and is also a major source of carbohydrates and proteins as well as other important phytonutrients (Habiyaremye et al., 2017). However, the protein content of millet is inadequate for infant feeding and limiting in some essential amino acids like lysine, which is required for children's growth (FAO, 1995; Friedman, 1996). Although millet has considerably high iron content, its bioavailability is low (Sihag, Sharma, Goyal, Arora, & Singh, 2015). In addition, millet has low levels of β‐carotene, vitamin C, zinc, calcium, sodium, and potassium (Queroz, 1991), which are required for child and infant feeding.

Thick porridge from millet flour is a major complementary food for infants and young children in most parts of Uganda (Temba, Njobeh, Adebo, Olugbile, & Kayitesi, 2016). The thick porridge from millet flour contains poor quality and inadequate protein and micronutrients required by weaning children. This is because it is nutritionally bulky and children have small volumes of the stomach. Therefore, they are not able to obtain adequate nutrients from the amount of porridge, and their stomachs are able to accommodate. Achieving a thin drinkable porridge requires the addition of copious amounts of water during preparation, which lowers the energy density (Adenike, 2012). However, processes such as sprouting/germination of millet grains activate endogenous enzymes that start modification of the millet grain constituents, causing changes in soluble sugars, protein, and fats that improve the nutritional quality, functional, and sensory properties of millet flour (Adenike, 2012). Consequently, starch (amylose and amylopectin) is degraded, resulting in a significant reduction in the viscosity and an increase in protein bioavailability during porridge preparation (Shaik, Carciofi, Martens, Hebelstrup, & Blennow, 2014). Germinating millet grains thus provides an easy way to improve the nutritional well‐being of weaning babies.

Despite the improvement in nutritional and functional properties brought about by germination of millet grains, overall the millet‐based diet remains inadequate in protein and micronutrients. Addition of vegetables and skimmed milk powder is a promising option to improve both the macro‐ and micronutrient profile and functional properties of millet flour. This is because vegetables are rich in β‐carotene, zinc, calcium, sodium, and potassium (Buttriss, 1989; Maass, 2014), while skimmed milk powder is rich in protein with balanced essential amino acids and high digestibility (Hoffman & Falvo, 2004). The aim of this study was therefore to develop a nutrient‐enhanced millet‐based composite flour incorporating skimmed milk powder and vegetables.

## 2 MATERIALS AND METHODS

### 2.1 Selection of raw materials

The materials used in this study included millet grains, pumpkin seeds, carrots, cowpea leaves, and skimmed milk powder. These raw materials were selected because of their superiority in the macro‐ and micronutrients essential for children aged 6–59 months. Skimmed milk powder has a high protein content with balanced essential amino acids and is stable during storage due to a low fat content (Hoffman & Falvo, 2004). It also increases calcium and iron bioavailability due to its high protein content (Gibson, Bailey, Gibbs, & Ferguson, 2010). Millet has high iron and calcium contents required for infant blood transportation and bone formation, respectively. In addition, it is a major source of energy for infant growth. Although iron in millet is less available, the addition of cowpea leaves and carrots and processing methods was suggested ways of improving the iron bioavailability (Christides, Amagloh, & Coad, 2015). Pumpkin seeds are rich in zinc with 7.5%–14% bioavailability of the zinc requirements (Glew et al., 2006), which is essential for child development, while carrots are rich in β‐carotene (Singh, Kawatra, & Sehgal, 2001), which is essential for immune development in infants aged 6–59 months. Cowpea leaves are rich in protein, iron, and calcium and have low levels of antinutrients compared with other protein‐rich plant sources (Chikwendu, Igbatim, & Obizoba, 2014).

### 2.2 Source of raw materials and laboratory reagents

Millet, pumpkin seeds, carrots, and cowpea leaves were purchased from Kisenyi‐Market, Kampala, Uganda, while skimmed milk powder was purchased from Pearl Dairies, Mbarara District, Uganda. All the materials were delivered to the laboratory at the School of Food Technology, Nutrition and Bioengineering, Makerere University for further processing. Laboratory reagents were purchased from Neo Faraday Laboratory Supply, Kampala, Uganda.

### 2.3 Preprocessing of millet grains

Millet grains were divided into two equal portions. One portion was germinated and the other roasted. Germination time was selected based on the sprouting/germination time of millet grains (1–2 days) (Saleh, Zhang, Chen, & Shen, 2013), while roasting temperatures were selected based on methods of Adebiyi, Adeyemi, and Olorunda (2002).

### 2.4 Preparation of vegetables

Cowpea leaves and carrots were washed with running tap water to remove surface soil. The cowpea leaves and carrots were blanched in a water bath (Grants Instrument Ltd, Shepreth, UK) maintained at 80°C for 10 min and 95°C for 5 min, respectively. After blanching, carrots were shredded using a hand grater with holes of a diameter of 0.6 cm. The shredded carrots and cowpea leaves were dried for 6 hr in an electric cabinet dryer (B.MASTER, Italy) set at 55°C. Dried carrots and cowpea leaves were then milled into fine powders using a locally fabricated hammer mill. Pumpkin seeds were cleaned and roasted at 140°C for 20 min using an infrared Food oven (GU‐6, New Zealand). Dry pumpkin seeds were blended into a fine powder using an electric blender (Lilaram Manomal and Sons, India).

### 2.5 Millet grains processing conditions

Germination time and roasting temperatures were varied as shown in Table 1. Regression analysis was used to determine the influence of germination time and roasting temperature on the nutritional quality of the millet flour. The protein digestibility, protein content, total phytates, and total sugars were expressed individually as functions of the germination time and roasting temperature. The data were fitted to the approximating linear model using Equations (1) and (2).

(1) $$Y=\beta_{0}+\beta_{1}X_{1},$$

(2) $$Y=\beta_{0}+\beta_{2}X_{2},$$

**Table 1:** Processing variables of millet grains and their levels

Variables	Symbol	Variable levels		
Germination time (hr)	*X*_1_	0	24	48
Roasting temperature (°C)	*X*_2_	80	100	140

where *Y *= Response function, *β*_0_ **= Constant (the intercept), *β*_1_ or *β*_2_ **= Coefficient of the linear effects, *X*_1_ and *X*_2_ **= Linear effects of germination time and roasting temperature, respectively.

### 2.6 Formulation of the composite flour

Nutri‐survey software, 2007 was used to estimate the optimum amounts of skimmed milk and vegetable powders to add to millet flour in order to meet the protein (13 g/day) and energy (1046 and 902 kcal/day for boys and girls, respectively) requirements for children of age 6–59 months (WHO, 2012). The details of the various combinations used to reach the optimum levels of millet flour, skimmed milk, and vegetable powders are indicated in Table 2.

**Table 2:** Formulations to obtain maximum levels of millet flour, skimmed milk, and vegetable powders used in Nutri‐survey software

Millet^a^ flour (g)	Skimmed milk powder (g)	Carrot powder (g)	Cowpea powder (g)	Pumpkin seed powder (g)	Energy (kcal/100 g)	Protein (g/100 g)
60	15	15	5	5	290	12.6
60	25	5	5	5	325	16.1
55	30	5	5	5	326	17.3
**65**	**20**	**5**	**5**	**5**	**323**	**14.8**
50	35	5	5	5	328	18.6

a Flour from millet germinated for 48 hr. The formulation in bold font was chosen as optimal and used in this study because it provided 113.9% and 35.8% of daily protein and energy requirements, respectively, for children aged 6–59 months.

### 2.7 Nutrient composition of millet flours

#### 2.7.1 Proximate composition and energy estimation

The millet and millet‐based composite flours were analyzed for moisture, crude protein, crude fat, crude fiber, and ash contents according to the method described by AOAC (2010). Carbohydrate was determined by difference, and energy content was determined using the Atwater factor (carbohydrate and protein values were each multiplied by 4 kcal/g, whereas fat values were each multiplied by 9 kcal/g).

#### 2.7.2 Determination of minerals

The amount of calcium, zinc, iron, magnesium, and copper in the composite flour was measured by an atomic absorption spectrophotometer according to the method of Hernández‐Urbiola, Pérez‐Torrero, and Rodríguez‐García (2011). Calibration equations were derived, and concentrations of calcium, zinc, iron, sodium, and potassium were expressed as mg/100 g.

$$\text{Concentration}\;\left(\text{mg}/100\;g\right)=\frac{((\text{Slope}\;\times \;\text{Absorbance})-y)\;\times \;D\;\times \;V}{\text{Sample weight (g)}\;\times \;10},$$

where *y* = intercept on *y*‐axis; *D* = Dilution factor; 10 =  a conversion factor from mg/kg to mg/100 g; *V* = volume of sample in ml.

#### 2.7.3 Determination of β‐carotene

The β‐carotene content of flour was determined according to Rodriguez‐Amaya and Kimura (2004). Carotenoid and the β‐carotene contents were expressed as μg/g and μg RAE, respectively.

$$\text{Carotenoid content}\;\left(\mu g/g\right)=\frac{A\;\times \;V(\text{ml})\;\times \;10^{4}}{A_{1\mathrm{cm}}^{1\%}\;\times \;p(g)},$$

where *A* = absorbance; *V* = total extract volume; *p* = sample weight; $A_{1\mathrm{cm}}^{1\%}$ = 2592 (β‐carotene extinction coefficient in petroleum ether).

$$\beta -\text{carotene}\;\left(\mu g\;\text{RAE}\right)=\frac{\text{Carotenoid content}}{12},$$

where 12 = retinol activity equivalent conversion factor, 1 RAE = 12 μg β‐carotene.

#### 2.7.4 Determination of total phytates

A modified method of Ijarotimi and Babatunde (2013) was used for the determination of phytate content in millet flour.

#### 2.7.5 Determination of protein digestibility

Protein digestibility was determined by the porcine pepsin method as adapted by Gomez, Obilana, Martin, Madzvamuse, and Monyo (1997).

#### 2.7.6 Determination of total sugars

Total sugars were determined according to the method described by Nielsen (2010).

### 2.8 Determination of physical properties of millet‐based composite flour

The bulk density, water, and oil absorption capacities of the millet flours were determined according to the method described by Onwuka (2005).

### 2.9 Determination of swelling capacity and solubility of millet‐based composite flour

The method described by Leach et al. (1959) was used to determine swelling power and solubility of the sample. The swelling power and solubility of the samples were expressed as %.

$$\text{Swelling power}\;\left(\%\right)=\frac{\text{weight of sediment paste (g)}\;\times \;100}{\text{weight of sample (g)}\;\times \;(100\;-\;\%\text{Solubility)}},$$

$$\text{Solubility}\;\left(\%\right)=\frac{\text{weight of soluble starch (g)}\times 100}{\text{weight of sample (g)}}$$

### 2.10 Pasting properties

Pasting characteristics of the porridge from the composite flour were determined with a Rapid Visco Analyzer (Perten Instruments AB, Kungens Kurva, Sweden) according to Ikegwu, Okechukwu, and Ekumankana (2010). Peak viscosity, trough, breakdown, final viscosity, setback, peak time, and pasting temperatures were read from the pasting profile with the aid of thermocline for windows software connected to a computer (Newport Scientific, 2001). The viscosity was expressed in centipoises (cP).

### 2.11 Contribution of porridge from millet‐based composite flour to RDA

Percentage contribution to recommended dietary allowance was expressed as % of RDA.

$$\%\text{RDA}=\frac{X}{Y}\times 100,$$

where *X* is the amount of nutrient analyzed and *Y* is the RDA for a given nutrient/variable.

### 2.12 Statistical analysis

All determinations were performed in duplicates and subjected to statistical two sample *t* test analysis of variance (ANOVA) using XLSTAT software version 2017 to determine variation between means. Significance variation was accepted at *p* < 0.05. Simple linear regression analysis was used to determine the influence of germination time and roasting temperature on the nutritional quality of the millet flour.

## 3 RESULTS AND DISCUSSION

### 3.1 Effect of roasting and germination on the nutrient content and quality of millet flour

The effect of roasting and germination on the nutrient composition of millet flour as predicted by the linear regression models is indicated in Table 3. Germination time had a positive linear effect on the protein content, total sugar content, and protein digestibility as well as a negative linear effect on the total phytates in millet flour. According to the results in Table 3, an increase in germination time significantly (*p* < 0.05) increased the protein content, protein digestibility, and total sugar content of millet flour by 0.03%, 0.79%, and 0.07%, respectively, and reduced the total phytates by 0.005 mg/g. Roasting temperatures had a positive linear effect on the protein digestibility and a negative linear effect on the total phytates, protein, and total sugar contents of millet flour. An increase in roasting temperature significantly (*p* < 0.05) reduced the total phytates by 0.003 mg/g, protein content, and total sugar content of millet flour each by 0.01%. However, an increase in roasting temperature increased the protein digestibility of millet flour by 0.34%.

**Table 3:** Effect of roasting and germination on the nutrient composition and quality of millet flour

Variable	No treatment (Intercept)	Treatment (*p*)		*R* ^2^
		Germination (*X*_1_)	Roasting (*X*_2_)	
Protein	9.3	0.03 (<0.05)	−0.01 (<0.05)	0.98
Protein digestibility	20.9	0.79 (<0.05)	0.34 (<0.05)	0.76
Phytates	3.9	−0.005 (<0.05)	−0.003 (<0.05)	0.97
Total sugars	2.2	0.07 (<0.05)	−0.01 (<0.05)	0.98

### 3.2 Optimal processing conditions of millet flour

**Figure 1:**
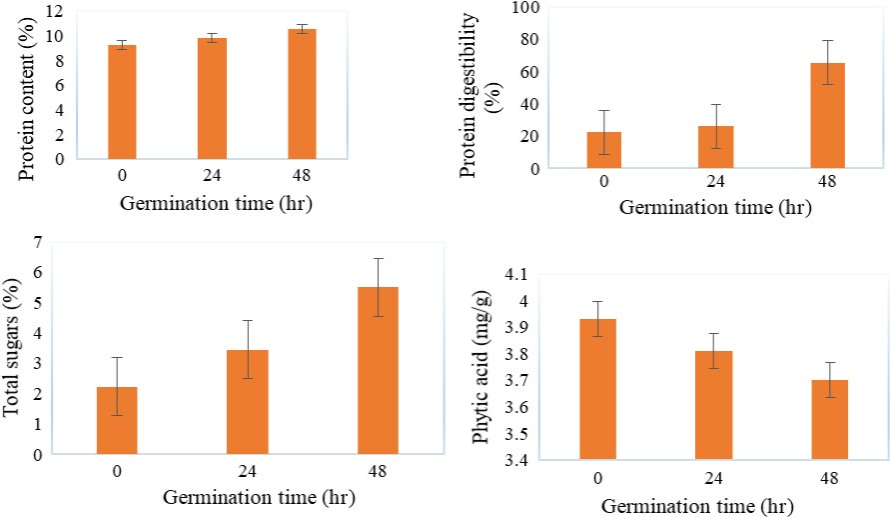
Effect of germination time on the protein content, protein digestibility, total sugars, and total phytates in millet flour

**Figure 2:**
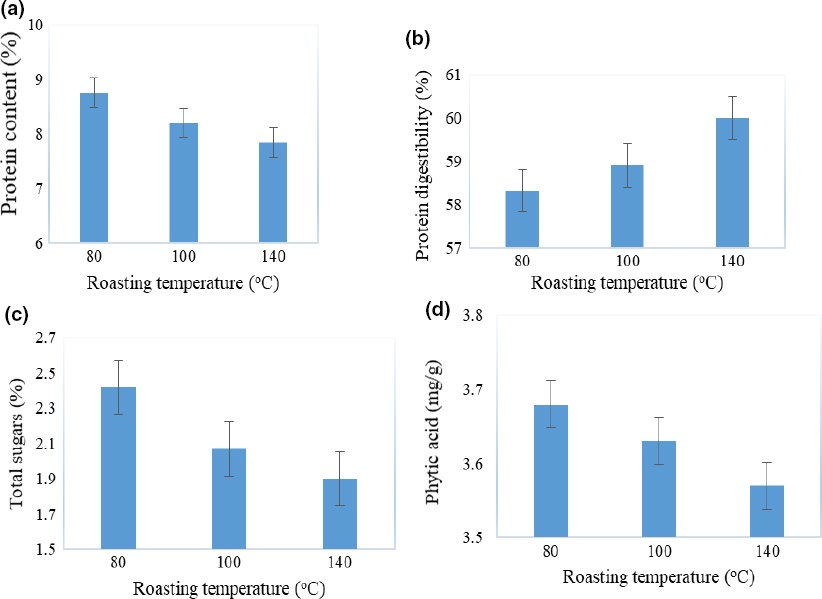
Effect of roasting temperature on the protein content (a), protein digestibility (b), total sugars (c), and total phytates (d) in millet flour

Figures 1 and 2 show the effect of processing conditions on the nutrient composition of millet flour. Figure 1 shows that germinating millet grains for 48 hr significantly (*p* < 0.05) increased the protein content (9.3%–10.6%), total sugars (2.2%–5.5%), and protein digestibility (22.3%–65.5%). Results in Figure 2 show that roasting millet at 140°C significantly (*p* < 0.05) reduced total sugars (2.2%–1.9%), protein content (9.3%–7.8%), and phytates (3.9–3.6 mg/g) but increased protein digestibility (22.3%–60.1%). The study findings indicate that germination for 48 hr resulted in millet flour with a high protein content (10.6%), protein digestibility (65.5%), total sugars (5.5%), and low levels of phytates (3.7 mg/g) and was therefore taken as the optimal procedure to process the flour that was used in the formulation of the composite flour. The increase in the total sugars during germination of millet grains is due to the hydrolysis of starch into shorter chain sugars by amylases. The results of this study agree with those reported by other researchers. Coulibaly and Chen (2011) reported a drastic increase in total soluble sugars (1%–13%) in foxtail millet germinated for 6 days.

The significant increase in protein content observed in flour from germinated millet grains is attributed to an increased water activity that activates hydrolytic enzymes (Nonogaki, Bassel, & Bewley, 2010) as well as compositional changes associated with dry matter loss following the degradation of other constituents (Abdelhaleem, El Tinay, Mustafa, & Babiker, 2008). The physical mechanisms and biochemical reactions that explain the observed increase in bioavailability of protein content during the germination process are strongly associated with the morphology of the finger millet seed (Hejazi & Orsat, 2016). In the finger millet, endosperm represents the largest portion of the grain, which consists of an aleurone layer and three distinct starchy sections: peripheral, corneous, and floury endosperms. The peripheral endosperm contains small and tightly packed cells of protein bodies that are embedded in fiber–starch–protein matrices (Belton & Taylor, 2002). The observed increase in the crude protein level during germination was the outcome of the activation of the plant α‐amylase (Traoré, Mouquet, Icard‐Vernière, Traore, & Trèche, 2004). The activity of α‐amylase resulted in the starch granules breakdown, which led to the release of the protein from the packed cells and consequently, increased its bioavailability. Findings from this study are in agreement with those of Chaturvedi (2015), who also reported a drastic increase in protein content from 12.5%–58.6% in foxtail millet germinated for 48 hr.

The increase in protein digestibility observed in flour from germinated millet grains is due to phytic acid hydrolysis, which is known to interact with proteins to form complexes as well as degradation of long protein chains by proteases whose activity increases during germination of cereals (Hassan et al., 2006; Mbithi‐Mwikya et al. 2000). Osman (2007) observed a similar trend in germinated Lablab beans.

Phytates form complexes with proteins, hence causing a detrimental impact by reducing protein digestibility and amino acid availability (Selle, Cowieson, Cowieson, & Ravindran, 2012). Reduction in phytates is due to increased phytase activity during germination (Azeke et al., 2011; Coulibaly & Chen, 2011). A decrease in total phytates (246–320 mg/100 g) of millet flour with an increase in germination time was similarly observed by Shashi, Sharan, Hittalamani, Shankar, and Nagarathna (2007).

Reduction in the total sugar levels and protein content during roasting of millet grains could be attributed to Maillard reactions and caramelization, which occur at higher temperatures (Baker et al., 2003). During roasting, sugars, and amino acids, in particular lysine because of its free ∈‐amino groups, undergo Maillard reactions, thus reducing protein and sugar contents (Gilani, Xiao, & Cockell, 2012) of millet flour. In addition, roasting reduces the protein content of millet flour due to protein denaturation (Kavitha & Parimalavalli, 2014).

### 3.3 Proximate composition of millet‐based composite flour

Table 4 shows the proximate composition of millet‐based composite flour on a dry weight basis. Results in Table 4 show that addition of vegetables and skimmed milk powders to the millet, significantly (*p* < 0.05) increased the crude protein, crude fat, crude fiber, ash, and gross energy of millet‐based composite flour by 7.3%, 2.1%, 2.5%, and 1.6%, respectively. These results further indicated that moisture content and carbohydrate content of the millet‐based composite flour significantly (*p* < 0.05) decreased by 1.1% and 9.9%, respectively.

**Table 4:** Proximate composition (%) of millet‐based composite flour on a dry weight basis

Sample	Moisture	Ash	Fat	Protein	CH_2_O	Energy (kcal)	Crude fiber
Millet‐based composite flour*	6.1^b ^± 0.1	4.2^a ^± 0.1	3.7^a ^± 0.0	15.3^a ^± 0.5	70.7^b ^± 0.1	378^a ^± 0.1	8.5^a ^± 0.3
Millet flour	7.2^a ^± 0.0	2.6^b ^± 0.0	1.6^b ^± 0.3	8.0^b ^± 0.1	80.6^a ^± 0.0	369^b ^± 0.0	6.0^b ^± 0.2

Notes

Values in the table are means of duplicate determinations ± standard deviations. Means in the same column with different superscripts are significantly different (*p* < 0.05).

*Millet composite flour contains millet flour, cowpea leaves, pumpkin seeds, carrots, and skimmed milk powders. CH_2_O = carbohydrates.

The decrease in the carbohydrate content of the composite flour is attributed to the dilution effect of skimmed milk and vegetable powders, which are low in carbohydrates. The increase in the crude protein content is attributed to the addition of skimmed milk and cowpea leaves powders. Findings of this study are in agreement with those of Kumar et al. (2013), who reported an increase in the crude protein content (13.6%–17.5%) due to the addition of skimmed milk powder and legumes to cereal flour. Increases in crude fat and fiber content of millet‐based composite flour may be attributed to the addition of pumpkin seed powder, which is reported to contain high fat (29%) (Soha, Sameh El‐SaftyAbd El‐Ghany, & Dalia, 2010) and crude fiber (31.5%) (Nyam, Lau, & Tan, 2013). Rico, Martín‐Diana, Barat, and Barry‐Ryan (2007) reported that food preparations enriched with dried vegetables have higher values of fiber. The reported crude fiber content was slightly higher than 5 g per 100 g recommended by the Codex Alimentarius for complementary foods. In case, the product is to be fed to malnourished children, and further modification will be required to reduce the fiber content to the required standard. The significant (*p* < 0.05) increase in the ash content of millet‐based composite flour is attributed to the addition of vegetables as they are reported to be rich in minerals. Findings from this study are in agreement with those of Mogra and Midha (2013), who observed an increase in ash content of germinated wheat flour enriched with green gram and spinach. The energy content of millet‐based composite flour significantly (*p* < 0.05) increased, as was to be expected from the increased fat content (Table 4). The results of the energy content from this study are in agreement with the findings of Balasubramanyam and Lokesh (2000), who reported that supplementary foods prepared from cereals and pulses provide 10%–30% proteins and 350–380 kcal energy.

### 3.4 Mineral and vitamin A (RAE) content of millet‐based composite flour

Table 5 shows the results of mineral and vitamin A content of millet‐based composite flour. A significant increase (*p* < 0.05) in zinc (2.1–4.2 mg/100 g), copper (0.5–0.9 mg/100 g), and calcium (143.6–667.8 mg/100 g) contents was observed in the millet‐based composite flour. The results in Table 5 further indicate that the increases in iron (3.4–3.6 mg/100 g) and magnesium (4.3–4.4 mg/100 g) were not significant (*p* > 0.05). The findings also indicate that the vitamin A content of millet‐based composite flour was significantly (*p* < 0.05) higher (641 μg RAE/100 g) than that of millet flour (15.5 μg RAE/100 g). The increase in vitamin A content of the millet‐based composite flour is attributed to the addition of carrot and cowpea leaves. Findings from this study were in agreement with those of Sadana, Bakhetia, and Aggrawal (2008), who reported an increase in vitamin A (1,021–1,322 mg/100 g) after supplementing germinated wheat and soy with carrot powder.

**Table 5:** Mineral (mg/100 g) and vitamin A (μg RAE/100 g) content of millet flour and millet‐based composite flour

Nutrient	Sample	
	Millet‐based composite flour*	Millet flour
Ca	667.8^a ^± 0.0	143.6^b ^± 0.0
Fe	3.6^a ^± 0.1	3.4^a ^± 0.3
Zn	4.2^a ^± 0.1	2.1^b ^± 0.2
Cu	0.9^a ^± 0.0	0.5^b ^± 0.0
Mg	4.4^a ^± 0.0	4.3^a ^± 0.0
Vitamin A	641^a ^± 0.1	15.5^b ^± 0.1

Notes

Values in the table are means of duplicate determinations ± standard deviations. Means in the same row with different superscripts are significantly (*p* < 0.05) different.

*Millet‐based composite flour contains millet flour, cowpea leaves, pumpkin seeds, carrots, and skimmed milk powder.

Addition of vegetable powders to millet flour boosted the mineral content because cowpea leaves are rich in zinc, iron, calcium, and magnesium, while pumpkin seeds are rich in zinc and copper (Barrett, Beaulieu, & Shewfelt, 2010; Vicente, Manganaris, Sozzi, & Crisoto, 2009). The findings from the analysis of the mineral contents of millet‐based composite flour are in agreement with those reported by Sadana et al. (2008), who observed that products prepared from germinated legumes had significantly higher values of minerals than products prepared from germinated wheat alone. Based on compositional analysis (Table 5), daily consumption of 100 ml porridge from the millet‐based composite flour will meet the RDA of children 6–59 months.

### 3.5 Contribution of mineral and vitamin A content of porridge prepared from the millet‐based composite flour toward RDA for children aged 6–59 months

Table 6 shows the mineral and vitamin A composition of porridge from millet‐based composite flour as a percentage of the recommended dietary allowances for children aged 6–59 months. Findings show that the porridge from composite flour contributed more than 100% of the required zinc (102.0%), copper (158.9%), and vitamin A (142.4%). However, the porridge from composite flour only contributed 35.8% of the RDA for iron in children aged 6–59 months. The results further indicate that the mean calcium (95.4%) contribution was closest to 100% of the RDA for children aged 6–59 months. The high contributions of zinc, copper, and calcium are due to high concentrations of these minerals as indicated in Table 5. Zinc, copper, and vitamin A are nontoxic in the body (Food and Nutrition Board, 1989), and therefore, their high levels in the millet‐based composite flour have no health concern. Therefore, adoption of the millet‐based flour and its proper preparation may greatly contribute to the reduction in mineral deficiencies among children aged 6–59 months.

**Table 6:** Contribution (%) of mineral and vitamin A content of porridge prepared from 100 g of millet‐based composite flour in 700 ml of water toward RDA for children aged 6–59 months^a^

Contribution to RDA	Ca	Fe	Zn	Cu	Vitamin A
Millet‐based composite flour^b^ (%)	95.4	35.8	102.0	158.9	142.4
Millet flour (%)	20.5	34.1	50.5	92.9	3.4
RDA (mg/100 ml)	700.0	10.0	4.1	0.6	450^c^

a The recommended levels of the nutrients considered adequate for most healthy children aged 6–59 months (Food and Nutrition Board, 1989).

b Millet composite flour contains millet flour, cowpea leaves, pumpkin seeds, carrots, and skimmed milk powder.

c μg/100 g while those without are in mg/100 g.

### 3.6 Contribution of energy, protein, and fat content of porridge prepared from 100 g of millet‐based composite flour in 700 ml of water toward RDA for children aged 6–59 months

Table 7 shows the contribution of millet‐based composite flour to the RDAs of energy, protein, and fat for children aged 6–59 months. The findings show that the protein contribution to the RDA reduced with an increase in the age of children. For children 0.5–1 year, the porridge from millet‐based composite flour would meet 109% of the RDA for protein, but it meets only 63.8% for children aged 4–6 years. Energy and fat contribution followed the same trend as that of protein, but all values were below 100% (Table 7).

**Table 7:** Contribution (%) of energy, protein, and fat content of porridge from 100 g of millet‐based composite flour in 700 ml of water toward RDA for children aged 6–59 months

Variable	Age group (years)	RDA	Contribution (%) of millet‐based composite porridge to RDA
Energy (kcal/day)	0–0.5	650^a^	58.2
	0.5–1	850^a^	44.5
	1–3	1300^a^	29.1
	4–6	1800^a^	21.0
Protein (g/day)	0–0.5	13^a^	117.7
	0.5–1	14^a^	109.3
	1–3	16^a^	95.6
	4–6	24^a^	63.8
Fat (g/day)	0–0.5	—	—
	0.5–1	—	—
	1–3	16.7^b^	22.2
	4–6	23.3^b^	15.9

a Food and Nutrition Board (1989).

b Alasfoor, Rajab, and Al‐Rassasi (2009).

The high contribution of the millet‐based composite porridge to RDAs for protein and energy is due to high concentrations of these nutrients in the composite flour (Table 5). In addition, the contributions of protein that are above the RDA are nontoxic to the body (Food and Nutrition Board, 1989; Nutrition Board and Institute of Medicine, N. A., 2011) because it was slightly above the protein requirement. However, it is recommended that protein intake should not be more than twice the RDA for protein (Food and Nutrition Board, 1989). Reduction in the contribution of energy, protein, and fat to the RDA with an increase in age is due to an increase in the body needs during growth. For example, energy is needed for metabolic activities and body maintenance, while protein is for growth and development in children. Fats are essential in the body for; calorie supply, brain development, absorption and transportation of vitamins A, D, E and K. In order to meet the protein, fat, and energy RDAs of the older children, intake of more than 100 ml of the millet‐based composite porridge is recommended.

### 3.7 The physical properties of millet‐based composite flour

Table 8 shows the physical properties of millet‐based composite flour. The oil absorption capacity (OAC) and water absorption capacity (WAC) of millet‐based composite flour were significantly (*p* < 0.05) higher than those of millet flour but bulk density did not change (0.6 g/ml) on the addition of vegetables and skimmed milk powder. OAC of the millet‐based composite flour increased from 59.2% to 77.9%, while WAC capacity increased from 117% to 225%.

**Table 8:** Physical properties of millet flour and millet‐based composite flour

Sample	Water absorption capacity (%)	Oil absorption capacity (%)	Bulk density (g/ml)
Millet flour	117.2^b^ ± 2.0	59.2^b^ ± 0.2	0.6^a^ ± 0.0
Millet‐based composite flour*	225.1^a^ ± 1.4	77.9^a^ ± 1.1	0.6^a^ ± 0.0

Notes

Values in the table are means of duplicate determinations ± standard deviations. Means in the same column with different superscripts are significantly (*p* < 0.05) different.

*Millet‐based composite flour contains millet flour, cowpea leaves, pumpkin seeds, carrots, and skimmed milk powder.

OAC is the ability of flour protein to physically bind fat by capillary attraction, and it is of great importance as fats act as flavor retainer and increase the mouthfeel of foods (Soria‐Hernández, Serna‐Saldívar, & Chuck‐Hernández, 2015). The increased OAC can be attributed to the high protein content of the millet‐based composite flour, which enhanced hydrophobicity by exposing more polar amino acids to the fat (Were, Hettiarachchy, & Kalapathy, 1997). This observation is consistent with the reports of Chandra, Singh, and Kumari (2015), who observed an increase in OAC of composite flours prepared by blending wheat flour with rice flour, mung bean flour, and potato flour from 146% to 156%. The values observed in this study were higher than those of sweet potatoes flour (10%–12%) (Choi and Yoo, 2008) but lower than those in lupin seed flour (167%) (Sathe et al., 1982).

The high WAC of millet composite flour is due to the high protein content resulting from the addition of cowpea leaf and skimmed milk powder as well as to the changes in the quality and quantity of proteins in the flour upon germination of millet grains (Sreerama, Sashikala, Pratape, & Singh, 2012). The higher protein content of the composite flour increased hydrogen bonding thus facilitating water binding and entrapment (Altchul and Wilcke, 1985). The high WAC of millet‐based composite flour gives it the advantage of being easily soluble in water while preparing porridge. The values of WAC obtained in this study were higher than the values reported for taro flours (red—180%; white—166%; and nive—150%) (Tagodoe and Nip, 1994), soybean flour (130%) (Lin et al.*,* 1974), fluted pumpkin seed flour (85%) (Fagbemi and Oshodi, 1991), and sweet potato flours (red—24% and white—26%) (Osundahunsi et al.*,* 2003), probably because of the high protein content of the millet‐based composite flour. However, the values obtained were in the range of those reported by Fasasi, Eleyinmi, and Oyarekua (2007), which were 226%–270% in processed millet flour samples.

Incorporation of vegetable and skimmed milk powders did not change the bulk density of millet composite flour (Table 8). This could be attributed to the low weight of the added powders in comparison with millet flour. In contrast, Edema, Sanni, and Sanni (2005) reported a decrease in bulk density of maize with increasing soy supplementation. This is probably because of the high weight of soy flour. The low values of bulk densities of millet composite flour make it suitable for high nutrient density formulations of foods.

### 3.8 Functional properties of millet‐based composite flour

#### 3.8.1 Effect of temperature on the solubility of millet‐based composite flour

**Figure 3:**
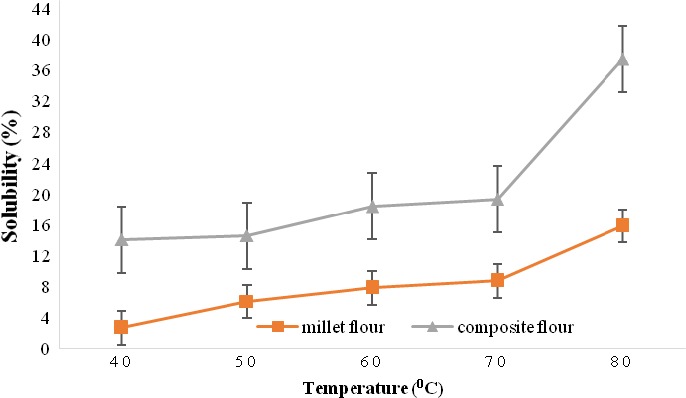
Solubility of millet flour and millet‐based composite flour at different temperatures

Figure 3 shows the solubility of millet flour and millet‐based composite flour at different temperatures. The results indicate that the solubility of the millet‐based composite flour was higher than that of millet flour at any temperature. These results further indicate that the solubility of millet flours generally increased with an increase in temperature from 14.0% to 37.4%. There was a gradual increase in the solubility of millet composite flour (14.0% to 19.3%) and millet flour (2.7%–8.7%) between 40 and 70°C, followed by a sharp increase between 70°C and 80°C (Figure 3).

According to Alcázar‐Alay and Meireles (2015) and Bolaji et al. (2014), increases in temperature allow starch granules to swell and eventually burst, leading to the leaching of amylose molecules into the water. Amylose forms hydrogen bonds with water molecules, hence the solubility of starch. The high solubility of millet‐based composite flour can be attributed to high sugar content as a result of germinating millet grains and the high protein content due to the addition of skimmed milk powder and cowpea leaves powder. The high sugar content might have favoured formation of hydrogen bonds and protein increased hydrophilicity of the flour, hence the high solubility (Alcázar‐Alay & Meireles, 2015).

#### 3.8.2 Effect of temperature on swelling power of millet‐based composite flour

**Figure 4:**
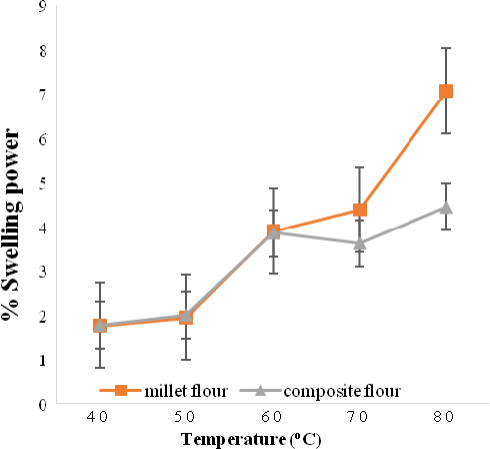
Swelling power of millet flour and millet‐based composite flour at different temperatures

Figure 4 shows results of the effect of temperature on the swelling power of millet‐based composite flour. The swelling power of the millet‐based composite and millet flours generally increased with an increase in temperature from 1.8% to 4.5% and 1.8% to 7.1%, respectively. Between 40 and 60°C, there was no significant difference in the swelling power of millet‐based composite and millet flours. Between 60 and 80°C, the swelling power of millet flour was higher than that of millet‐based composite flour.

The general increase in swelling power of flours is due to gelatinization of starch as a result of heating in the presence of excess water. This could have resulted in water diffusing into the starch granules (Jiménez et al.*,* 2012). The starch granules substantially increased in size due to hydration of the amorphous phase causing loss of crystallinity and molecular order (Jiménez et al.*,* 2012). At low temperatures (40–50°C), starch granules are less soluble in water due to the hydrogen bonds and crystallinity of the molecule, thus resulting in the low swelling power observed in both samples. Between 50 and 80°C, which is the porridge cooking temperature, the size of starch granules increased substantially, breaking the molecules and consequently leaching the amylose to form a three‐dimensional network and increased the paste's viscosity (Sarkar, Thapar, Kundnani, Panwar, & Grover, 2013). The trend observed in this study was also reported by Adebowale, Adeyemi, and Oshodi (2005) for red sorghum flour. The swelling power of the millet‐based composite flour was lower than that of millet flour due to the germination of the millet grains. Amylases break down starch during germination of millet grains, thus reducing the swelling power of the resultant flour. The low swelling power of the millet‐based composite flour makes it suitable as a weaning food as it will result in porridges of low viscosity.

### 3.9 Visco‐elastic properties of the millet‐based composite flour

**Figure 5:**
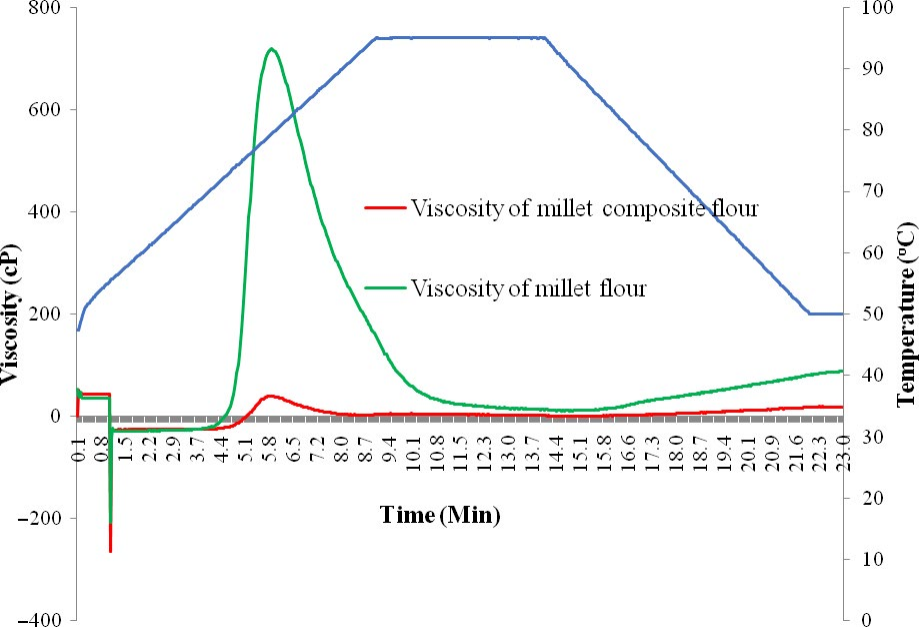
Rapid Visco‐Analyzer pasting curves for millet flour and millet‐based composite flour

Figure 5 shows the visco‐elastic properties of millet‐based composite flour. The results indicate that millet‐based composite flour recorded a lower peak (38 cP) and lower final viscosity (17.7 cP) compared to the 712.7 and 88 cP recorded for the flour from nongerminated millet grains. The composite flour recorded lower pasting time (2.6 min) and temperature (55.7°C) compared to that of flour from nongerminated millet grains at 3.3 min and 67.9°C, respectively.

The low peak viscosity and final viscosity observed in millet composite flour may be a result of modification of millet starch by amylases during germination of millet grains. Final viscosity is the change in the viscosity after holding cooked starch at 50°C, and it is a measure of the stability of the granule. Final viscosity indicates the ability of starch to form a viscous paste or gel after cooking and cooling (Alcázar‐Alay & Meireles, 2015; Iwe & Agiriga, 2014). The results in Figure 5 indicate that millet‐based composite flour had a lower final viscosity (17.7 cP) than millet flour (96.2 cP). This is nutritionally beneficial in infant formulas (Journal & North, 2014), as a less viscous porridge is a better weaning food for children.

Peak viscosity is an indicator of the thickening behavior and water holding capacity of starch (Alcázar‐Alay & Meireles, 2015; Iwe & Agiriga, 2014). The low peak viscosities observed in millet‐based composite flour might be attributed to millet starch modification by the amylase enzymes during germination. The low peak viscosity exhibited by millet‐based composite flour is suitable for products requiring low gel strength and elasticity. This property makes the millet‐based composite flour a suitable weaning food for children because they require porridge with low viscosity.

The setback or viscosity of the cooked paste is the viscosity after cooling to 50°C. The extent of increase in viscosity on cooling to 50°C reflects the retrogradation tendency (Chibuzo, 2012), a phenomenon that causes the paste to become firmer and increasingly resistant to enzyme attack (Horstmann, Lynch, & Arendt, 2017). It thus has an effect on digestibility. Higher setback values are synonymous to reduced paste digestibility (Shittu & Adedokun, 2010), while lower setback during cooling of the paste indicates a lower tendency for retrogradation and subsequently higher digestibility (Sandhu, Singh, & Malhi, 2006). The low setback value (16.67 cP) for the millet‐based composite flour indicates that its paste would have a higher stability against retrogradation (Mazurs, Schoch, & Kite, 1957) than millet flour whose setback value was 77 cP. It also implies that the porridge when consumed by children will be easy to digest.

The pasting temperature of millet‐based composite flour (55.7°C) was significantly (*p* < 0.05) lower than that of millet flour (67.9°C). The pasting temperature provides an indication of the minimum temperature required for cooking the flours (Shimelis, Meaza, & Rakshit, 2006). The low pasting temperature of millet‐based composite flour implies that less energy is required for cooking porridge from millet‐based composite flour than for flour from nongerminated millet grains.

## 4 CONCLUSION

Germination of millet grains and incorporation of skimmed milk and vegetable powders resulted in a nutrient‐enhanced composite flour with improved functional properties. Scaling up production of this composite flour can contribute toward improving the nutrition of children in developing countries.

## CONFLICT OF INTEREST

The authors declare no conflict of interest.
